# Therapy resistance/chronicity, their determinants and potential solutions

**DOI:** 10.1007/s00406-020-01101-6

**Published:** 2020-02-13

**Authors:** Hans-Jürgen Möller

**Affiliations:** Universitätsklinik der LMU München, Nussbaumstr. 7, 80336 München, Germany

The current volume is indeed directed to clinicians and their everyday clinical work. It covers several psychopharmacological issues of great clinical relevance: therapy resistance, chronicity, side effects. All three of them belong to the general topic of difficult-to-treat patients. In this editorial, I will focus on those papers which focus on therapy resistance/chronicity.

In this context, the French population-based study (Panes et al.) on the use of Benzodiazepines is of great importance. She comes to the conclusion that the Benzodiazepine use was not in accordance with international and French guidelines in 30% of new hypnotic users and 20% of new anxiolytic users. Benzodiazepine use not in accordance with guidelines was defined by the authors as the concomitant dispensation of several benzodiazepines, the dispensation of benzodiazepine treatment over a longer period than recommended (in most guidelines no longer than 4 weeks), considering that the French recommendations distinguish between the time limitation for hypnotic (4 weeks) and anxiolytic benzodiazepines (12 weeks), and a new dispensing within the 2 months following the end of previous treatment of maximum recommended duration. Associated characteristics of non-compliance with the guideline recommendations were older age, treatment initiation by a psychiatrist, the presence of chronic disease, hospitalisation or another psychotropic treatment. All these background factors demonstrate that these are not the average patients of practitioners, but apparently difficult-to-treat patients.

This result is not unexpected and fits to the experience in other European countries and worldwide. Does this indicate an un-reflected prescription pattern of the doctors or a low-dose dependency in a large subgroup of patients or does it mirror the need of patients suffering from chronic sleep and anxiety disorders. The apparent need for long-term medication, which is the message of the data, and the respective background factors might be seen as consequence of the problem that the other indicated medications, such as e.g., the SSRIs for the treatment of anxiety disorders, are in many cases, not sufficiently efficacious [1]. Similarly, sleep disturbances as well as anxiety treated with benzodiazepines might be a consequence of other psychic disorders, for which the specific medication such as antidepressants or antipsychotics is not sufficient enough to reduce all respective symptoms [2–4]. Doctors or patients in such situations might look for other solutions, although they are unspecific for the respective disorder. Also, the special problem of mixed anxiety depression should be considered in this context [5].

The paper (Panes et al.) on Clozapin gives some further hints into problem of therapy resistance. The chart review on long-term treatment with Clozapin reports important aspects of therapy resistance/chronicity in schizophrenia and the related response/nonresponse trajectories 40–70% of patients on Clozapine have persistent psychotic symptoms (ultra-treatment resistant schizophrenia, UTRS). Of those patients who were diagnosed UTRS after about 10 months (mean duration) of treatment with Clozapine 87% remained unresponsive after about 7 years (mean duration), only 13% became responsive. Thus, even with Clozapine, which has the indication for treatment-resistant schizophrenia, we are faced with the problem that a large subgroup of patients is so treatment resistant that Clozapine as monotherapy is not sufficient efficacious to overcome the problem. Combination strategies or even better new medications are necessary. But there is only weak evidence for combination strategies and unfortunately, a new powerful antipsychotic for these cases is not on the horizon.

As mentioned before, combination treatment with antipsychotics needs further evaluation. Thus, the paper (Schmid-Kraepelin et al.), describing the design of a three-arm RCT comparing the combination of Olanzapine with Amisulpride vs. each compound as monotherapy, goes in the right direction. However, the study does not focus on treatment-resistant patients, but on acutely ill schizophrenia patients.

Treatment resistance can be induced by different risk factors. Among others, pharmacokinetic may play a relevant role [6] and thus pharmacokinetic studies are needed. In this context, the study (Kiss et al.) on the phenoconversion of CYP2D6 by inhibitors and how this modifies aripiprazole exposure is of great interest.

It is well known that treatment resistance is a huge problem in the treatment of depression [2], on an average, 30% of patients do not achieve response and 50% no remission. For decades, the only solutions to overcome this were different combination and augmentation therapies and last but not least ECT. With the inclusion of some second-generation antipsychotics such as quetiapine and aripiprazole as add-on treatments to a pre-existing antidepressant treatment, the augmentation strategies were enriched. But nevertheless, there is need for other innovative solutions. In the recent years, the infusion therapy with ketamine, an antagonist at the NMDA-receptor, in depression gave striking results: an immediate resolution of the depressive symptoms, including suicidality. Recently, this off-label use of the anesthetic Ketamine was completed by the license authorisation of an intranasal spray of Esketamine in the indication of therapy-resistant depression (defined by a sequence of unsuccessful treatment with two different antidepressants). After the positive decision of the FDA EMA followed some weeks ago. Thus, we will have this innovative approach soon available on the European market. This gives hope for an improved depression treatment and raises expectations that the prevalence of therapy refractory depression (defined by no response even after augmentations strategies, etc.) in consequence might be reduced and/or that even for these patients, Esketamine will be evaluated with a positive outcome. Although Ketamine/Esketamine is not a new compound, the goal-oriented development of this anti-glutamatergic approach, enables the use of a new pharmacological mechanism for depression treatment. This innovative development hopefully opens the doors for other compounds with the same or similar mechanism. Unfortunately, the glutamatergic approach in treatment-resistant schizophrenia, in case of the glycine reuptake inhibitor, Bitopertin preferentially oriented towards negative symptoms, was not successful (Möller et al. [7], as generally the whole glutamatergic approach (among others with compounds such as e.g., metabotropic glutamate receptor agonists) in schizophrenia, which demonstrated no or only low efficacy.
